# The roles of short-term plasticity and synaptic weights in self-organized criticality

**DOI:** 10.1186/1471-2202-14-S1-P192

**Published:** 2013-07-08

**Authors:** Hoon-Hee Kim, Jaeseung Jeong

**Affiliations:** 1 Department of Bio and Brain Engineering, KAIST, Daejeon, South Korea

## 

Neuronal avalanches represent one important way in which the network dynamics of neural systems may be propagated. Experiments utilizing multi-channel recordings of neural cultures have shown that the sizes of neuronal avalanches follow a power-law distribution, which is a key feature of a self-organized criticality [1]. Although self-organized criticality provides certain advantages for the processing of neural information in neural networks [1-3], the underlying mechanisms of these dynamics remain unclear. One hypothesis is that synaptic plasticity is the major prerequisite for self-organized criticality [4,5]. In the present study, we focused on the roles of short-term plasticity and the relationship between synaptic weights and self-organized criticality.

We simulated neural networks consisting of 300 simple integrate-and-fire neurons with all-to-all connections. Each synapse had depressive short-term plasticity and random initial synaptic weights. Small external currents were injected into individual neurons that were randomly selected at each step, and the sizes of the neuronal avalanches at each step were recorded. After approximately two million steps, the synaptic weights were altered by short-term plasticity, the sizes of the neuronal avalanches followed a power-law distribution, and a pattern of converging exponents was observed.

To investigate the effects of synaptic weights on self-organized criticality, neural networks were re-simulated without short-term plasticity; instead, the synaptic weights from the above simulation, which had been altered by short-term plasticity, were used. In this simulation, the sizes of the neuronal avalanches did not follow a power-law distribution, and the exponents of the power function always remained between -1.2 and -1.9. The same results were observed for randomly connected neural networks.

Although the synaptic weights of the simulated neural networks adjusted when the neural networks exhibited a self-organized criticality, neuronal avalanche size did not follow a power-law distribution when short-term plasticity was turned off. These results show that the key mechanism for self-organized criticality is synaptic plasticity rather than synaptic weights. Therefore, it is important that short-term plasticity be maintained in neural networks to ensure self-organized criticality.
